# Examining racism in health services research: A disciplinary self‐critique

**DOI:** 10.1111/1475-6773.13558

**Published:** 2020-09-25

**Authors:** Rachel R. Hardeman, J'Mag Karbeah

**Affiliations:** ^1^ Division of Health Policy & Management University of Minnesota School of Public Health Minneapolis Minnesota

Racial disparities in health have existed in the United States for centuries.^1^ In 1899, WEB Du Bois noted the prevalence of poor health among black people, describing it as an important indicator of societal racial inequality.^2^ Black Americans continue to have substantially worse health and shorter life expectancies than their white counterparts.^1^, ^3^ Particularly in health services research, evidence of black‐white disparities in health and health care costs have been acknowledged for decades. However, much of this work has been divorced from the social context of deeply seated racial oppression (read: racism) that has created it. This dangerously incomplete view of disparities often fails to evoke racism as the fundamental cause of these injustices.^4^ By separating health disparities from racism, we fail to recognize disparities as inequities—that is avoidable injustices. Instead, we focus on individual differences rather than the systems and structures that uphold and replicate them.

Disciplinary self‐critique, a tenet of public health critical race praxis (PHCRP), helps a discipline shine a light on itself from within in order to understand how its norms may inadvertently buttress inequities either within the discipline or in society at large.^5^ PHCRP also defines the term “outsiders within” as people who are members of a field but often marginalized within it because of their social identity. We, the authors of this commentary, represent the outsiders within. We understand and appreciate the discipline of health services research, its strengths and visions. We care deeply about how health services research can and should address health inequities and propel our discipline toward more equitable realization of its mission to produce new knowledge about the structure, processes, and effects of health services for individuals and populations. This commentary will interrogate the ways we as health services researchers pose our research questions, create methodological approaches, and interpret our findings. We, the authors, hope that this commentary will serve as a disciplinary self‐critique that will expose how our disciplinary practices are steeped in white supremacy. This commentary asserts that without acknowledging shortcomings within our discipline, we cannot identify solutions to the most vexing health equity issues in our field.

## 1 RACISM, WHITE SUPREMACY, AND HEALTH SERVICES RESEARCH

Structural racism lies underneath, all around, and across society. It refers to the normalization and legitimization of an array of dynamics—historical, cultural, institutional, and interpersonal—that routinely advantage whites while producing cumulative and chronic adverse outcomes for black people.^6^ Structural racism encompasses (a) history, which lies underneath the surface, providing the foundation for white supremacy in this country; (b) culture, which exists all around our everyday lives, providing the normalization and replication of racism; and (c) interconnected institutions and policies, they key relationships and rules across society providing the legitimacy and reinforcements to maintain and perpetuate racism.^6^ To produce antiracist research and to achieve health equity, we must acknowledge the influence of white supremacist ideologies within our discipline. However, our traditional notions of white supremacy keep us focused on hate groups and vulgar language rather than a culture and ideology born from the premise of black inferiority and false notions of race as biological that have permeated the ways in which we conduct our research. As a result, a white racial frame—the overarching worldview that encompasses important racial ideas, terms, images, emotion and interpretation, and lens by which white supremacy is perpetuated—then legitimizes structural racism by providing a narrative, belief system and worldview that upholds and sustains it.^7^ Our long‐held belief systems drive the research questions we ask, the methods we employ, and the interpretation of our research findings. By interrogating it head on, we have everything to gain in the fight for racial health equity and the production of antiracist research.

A disciplinary self‐critique of our:

1. *Research questions*. Predominant notions about race shape the way we as health services researchers frame our research questions. For example, research questions are often phrased as “what causes Black people to have so many disadvantages compared to whites; and, what forces are at work?” This question or variations of it are widely used and accepted among health services researchers and at first look appear innocuous and even virtuous. In reality, this type of question reinforces racism and white supremacy, suggesting a black deficit and subtly reinforcing a narrative and a story that is privileged over other stories. This framing retells the story of black people engaging in poor health behaviors; sometimes, these research questions highlight that black people have limited access to resources. These narratives are told over and over again. Rarely are research questions centered on “Why do Black people have stellar outcomes when compared to whites for x disease?” or “What aspects of Black social networks help individuals diagnosed with chronic diseases succeed?” Even when our research questions acknowledge that health is not solely the result of individual action, we fail to think of research questions that center black successes and triumphs. Thus, seemingly neutral research questions are not neutral. We must wonder if the research question “what causes white people to have so many advantages compared to Black people; and what forces are at work?” elicit similar responses. Reframing how we as health services researchers situate our research questions is important and necessary. For instance, early research on black maternal and infant mortality rates in the United States explored questions focused on the role of diet, activity, smoking, and drug use as the fundamental cause of observed disparities which frequently led to conclusions that upheld biological and/or behavioral failings of black birthing people.^8^ Based on the framing of these questions, researchers often concluded that individuals who do not access prenatal care or experience adverse outcomes only face these realities as a result of their personal actions and decisions.^9^, ^10^, ^11^, ^12^ By reframing the question in terms of structural failures and histories of exclusion and disenfranchisement, we learn that structural inequities such as racial discrimination within our education system, residential segregation, and environmental racism contribute to the racial inequities in black maternal mortality, inequities that exist even and when controlling for access to prenatal care. The fact that the hospitals serving majority‐black birthing populations perform worse on nearly every maternity care indicator^13^, ^14^ is not random—these hospitals reflect histories of racial discrimination, residential segregation, and systemic disinvestment.^15^
2. *Methodological approaches*. As a discipline, health services researchers have often elevated large quantitative datasets as the penultimate source of objectivity and the source of empirical fact. However, when we apply public health critical race praxis^16^ methodological approach, we see that our methods are fundamentally flawed because they rarely identify, name, and interrogate the influence of white supremacy, the white racial frame, and structural racism. If we know that racism causes health inequities, we must consider both developing better ways to measure systems of inequity and name racism as the central concept that racial categories attempt to measure if we continue to use racial categories in our analyses. Our methods, however, scientifically rigorous, are rarely objective. Our current analytic methods reflect the white racial framing of society in two fundamental ways: (1) We often incorrectly present racial categories as immutable biological fact when we fail to acknowledge that racism not race causes observed disparities^17^; and (2) we replicate society's white supremacist hierarchy. In our quest to understand disparities, we insert race variables into models and analyses without interrogating what these variables measure. Few researchers question why they control for race within their models or how having a racialized identity impacts their outcome of interest.^18^
  Our methodologies also often replicate white supremacist framing by making whites the dominant group to which we compare all other populations. Researchers rarely question why whites are the dominant group within their research or even if white outcomes are a desirable standard for populations to strive toward. Considering within‐group analyses or selecting a different comparison group may reveal new knowledge about the structural and social inequities at play.

- *Interpretation of findings*. There is often a conflation of race with racism in the interpretation of our research findings. By reporting findings such as black people were more likely to die from hypertension or black people do not access prenatal care, we are at best stating a disparity (difference) based on race and at worst suggesting that race (phenotype) dictates one's chances of survival, health, and well‐being. This may lead many down the path of biologic assumptions about race rather than understanding its operation as a social and political construct. An interpretation of findings must begin with the public health critical race praxis tenet referred to as “primacy of racialization”—the idea that our tendency is to attribute effects to *race* rather than to *racialization or racism*. Rather our interpretation of findings must include an interrogation of root causes (racism and white supremacy) and a consideration for how they mold the social determinants of health leading both to an unequal distribution of disease and well‐being (eg, increased asthma rates in neighborhoods with poor quality housing and increased environmental exposures) as well as psychosocial stressors and unhealthy behaviors.

## 2 THE EMANCIPATION OF HEALTH SERVICES RESEARCH

White supremacy and thus the white racial frame are so embedded within our unconscious thought that we have grown to accept the white‐dominant narrative as fact and even at times refer to it as objective science.^19^, ^20^ Dominant narratives refer to the stories told and retold within our society that serve the interest or ideologies of one dominant social group (ie, individuals seen as white). We often believe that these narratives are true because they have been normalized through repetition or because people in positions of power tell us they are true.^21^ Unconsciously, we often invest in these narratives and they can come to define our culture, values, and even our identities—which makes them particularly difficult to interrogate. A historical example of a dominant narrative would be the claim that the abolition of slavery would be disastrous for enslaved people because they could not adequately care for themselves—a narrative that was so widespread it was espoused by prominent medical professionals such as Drs. Benjamin Rush and Samuel Cartwright.^22^, ^23^ A contemporary example of a dominant narrative would be the claim that America is a meritocracy where success depends only on hard work and determination.

An emancipation from the dominant frame of whiteness is vital to efforts to eliminate health inequities and to become better health services researchers. Considering that today we face the result of centuries of well‐buttressed racial exploitation and oppression, major change will not come easily. As health services researchers, we must commit ourselves to the emancipation of our research from white supremacy. The first step in doing so requires us to make the white racial frame visible through deframing and counter‐framing. Deframing involves consciously taking apart and critically analyzing health inequities within the context of the white racial frame. Reframing refers to the acceptance and creation of a new frame with which to replace it.^7^ Disciplinary self‐critique^24^, ^25^ is an important first step in antiracist counter‐framing that can be employed in academic research. We cannot build a more equitable and just future using the same white supremacist tools that were used to create the systems of disadvantage that we seek to dismantle. The critical first steps have been laid out for us by several interdisciplinary scholars dating back to the beginning of the 20th century. We offer four readings as a starting place for disciplinary self‐critique: The Souls of Black Folk^26^; Critical Race Theory, Race Equity, and Public Health: Toward Antiracism Praxis^16^; Structural Racism and Supporting Black Lives—The Role of Health Professionals^27^; and On Racism: A New Standard for Publishing on Racial Health Inequities^28^ Finally, our research must be community‐engaged and, when possible, community‐led. Any research question that seeks to interrogate racial inequity must center the voices and experiences of those most impacted by the issue or outcome being studied.^29^

This commentary represents a first step in shining the light from within. In health services research, prevailing ideas about race have formed our early scientific research, but because investigators are unlikely to critique their relationships to their racialized social contexts, they lack the ability to perceive the influence of racism in their work.^25^ Even much of the race‐critical literature fails to call out specifically nor analyze who controls these major institutions and foundations, leaving white supremacist ideology unnamed and ever‐present. Indeed, new views and new ideas are regularly screened for conformity by those in power—decision makers in academia from journal editors and grant funders—to ensure that scholarship fits into accepted paradigms within the discipline.^30^

Another aspect to counter‐framing that builds upon the need to critically analyze the research questions in health services research is evaluation of the question of *whose evidence do we consider to be real?* In the academy broadly and in the field of health services research specifically, there is implicit agreement regarding who is able to generate evidence, and by extension, whose evidence is real and whose is not. The simple exclusion of black people in the academy and in health services research specifically has created a space in which few black people have an opportunity to contribute to the academic evidence base on health equity.^31^, ^32^, ^33^ The marginalization of black scholars in the academy has allowed for research to be produced and a body of evidence to be created that does not always authentically represent the lived experience of those closest to the topic of inquiry. Furthermore, those black or otherwise marginalized voices that do make it into the academy are often urged (both explicitly and implicitly) to take on the privileged narrative of their mentors, role models, and the academy writ large during their training—ultimately being socialized through the white racial frame. Simultaneously, these scholars are forced to reckon with the reality that research focused on health disparities so often yields criticism about its rigor and validity as science.^34^

We are now, however, in a moment in which the path forward compels us to change. We as health services researchers must emancipate ourselves from the dominant white supremacist framing that has touched every aspect of our science. We must strive to make what has for so long been invisible in health services research visible—there are lives that depend on it.
